# Fixing a fractured arthrodesed hip with rapid prototype templating and minimal invasive plate osteosynthesis

**DOI:** 10.1016/j.tcr.2015.10.005

**Published:** 2015-11-14

**Authors:** Christian Fang, Benjamin Fang, Tak-Man Wong, Tak-Wing Lau, Terence Pun, Frankie Leung

**Affiliations:** a Department of Orthopaedics and Traumatology, The University of Hong Kong, 5/F Professorial Block, Queen Mary Hospital, Pokfulam, Hong Kong, China; b Department of Radiology, H1, Queen Mary Hospital, Pokfulam, Hong Kong, China; c Shenzhen Key Laboratory for Innovative Technology in Orthopaedic Trauma, The University of Hong Kong-Shenzhen Hospital, Shenzhen, China

**Keywords:** Hip fusion, Fracture, Plating, Minimal invasive, 3D printing

## Abstract

**CASE:**

We present an elderly lady with an intertrochanteric fracture of a previously fused hip. A 3D printed model of her pelvis and femur was used for implant templating before surgery. Minimal invasive fixation was performed with a spanning reversed distal femur locking plate without the need for removal of the previous implant. Multiple long locking screws were placed in the supra-acetabular region. The patient had union in 4 months, return to function and no complication.

**Conclusion:**

The technique allowed us to optimize implant selection and insert screws safely at difficult trajectories using minimal invasive surgery.

## Introduction

Arthrodesis of the hip is a treatment option for painful chronic hip conditions. While it is much less often performed in the developed world with the popularization of total hip replacements (THR) [2], previous patients are usually functional in the long term [1]. Complications such as long lever arm fractures and adjacent joint disease after fusion are occasionally encountered which require follow-up care [3].

A fractured arthrodesed hip is rare. In literature, we found four isolated reported cases each using a unique surgical treatment. They included the use of open reduction with dual plating [4], combined plating and cannulated screws [5], short segment retrograde nailing through the subtrochanteric region [6] and cephalomedullary nail fixation [7]. In an osteoporotic elderly, we were sceptical about the extent of dissection or stability with these methods and sought for an alternative way.

Rapid prototyping is increasingly popular and available in the healthcare industry. In the musculoskeletal field, the technology is applied in patient specific instrumentation [8], [9], implant templating [10], surgical training [11] and tissue engineering [12]. The technology gave us a positive experience illustrated by this case report. Patient consent has been obtained for publishing this case.

## Presentation of the case

**Fig. 1:**
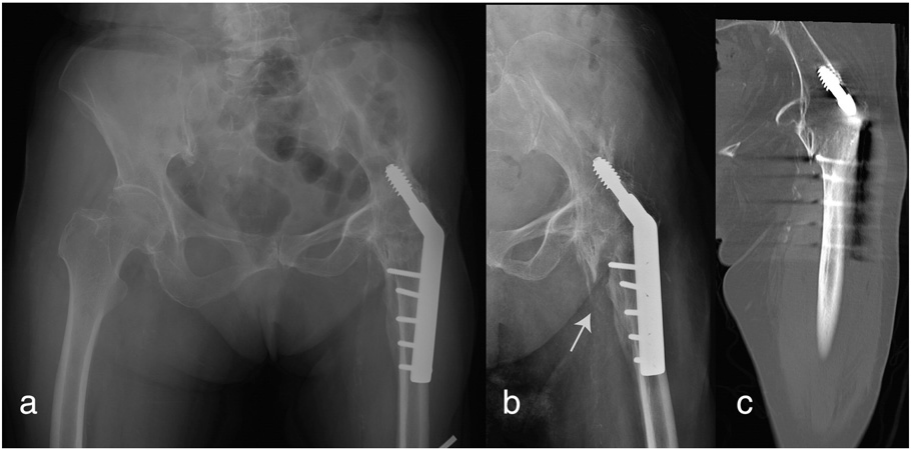
a) Pre-injury radiograph of an 88-year-old lady showing solid arthrodesis of the left hip with a DHS. b) X-ray showing an intertrochanteric fracture after injury. c) Reformatted coronal CT scan revealing the fracture in the trochanteric region with suspected loosening of the DHS lag screw.

We encountered an 88-year-old lady with a history of left hip tuberculosis managed with surgical fusion 16 years ago. The patient was an indoor walker with the need for a quadripod. She was admitted after a low energy fall and complained of severe mechanical pain at her left hip. X-rays revealed a mildly displaced intertrochanteric fracture at her previously fused hip. A DHS was previously used for the fusion surgery, and the lag screw which was previously fixed to the ilium bone appeared to have loosened (Fig. 1).

**Fig. 2:**
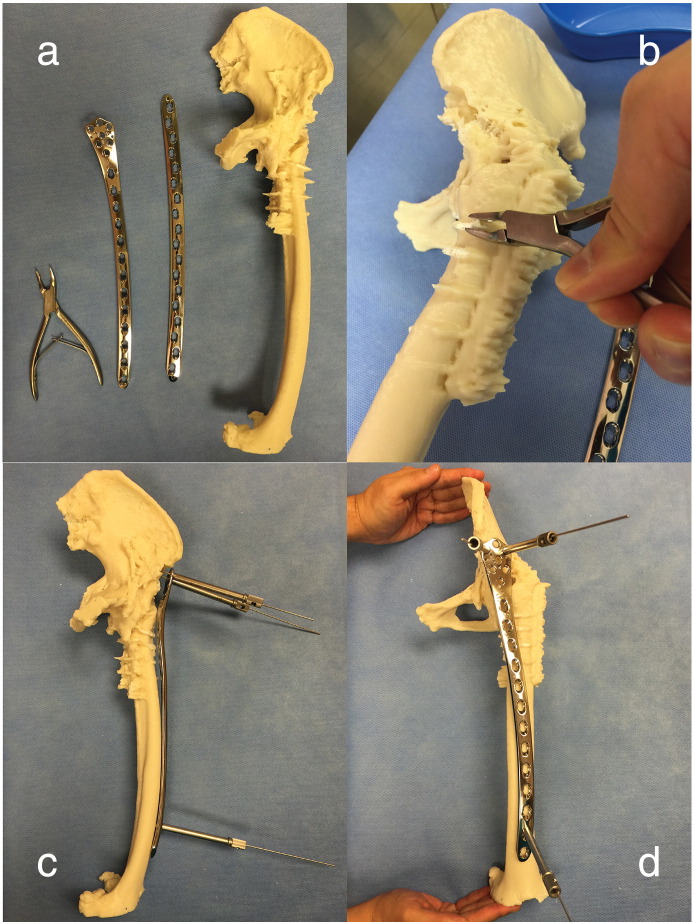
a) The 3D printed model was templated against a 4.5/5.0 mm curved broad LCP and a contralateral distal femur LCP. b) Artefacts from CT scan on the model were removed with a Rongeur to allow better contouring. c) Simulated preliminary fixation using Kirschner wires in the iliac oblique and d) obturator oblique views.

A CT scan was performed to confirm the location of the fracture and morphology of the fused iliofemoral segment. 0.5 mm cuts were obtained from the pelvis to distal femur. A 3D printed model was created from the volumetric CT data using a low cost thermoplastic (Acrylonitrile-Butadiene-Styrene) (Fig. 2). The 3D printing process took around half a day. This material was non-sterilisable under autoclaving temperatures but was deemed cost effective for the current purpose.

The model was created in 1:1 scale. Imperfections in the 3D model from radiological artefacts were trimmed away using a Rongeur. Two different types of long locked compression plate (LCP) for the femur were evaluated in preoperative templating. The reversed contralateral distal femur 4.5/5.0 mm LCP (DePuy Synthes, Solothurn, Switzerland) was found to offer the best fixation trajectories when bent multiple times to fit on the anterolateral supraacetabular region. Trial fixation with Kirschner wires was performed in the plastic model to ensure that the screw trajectories did not penetrate the greater sciatic foramen or the ilium tables.

A minimal invasive plate osteosynthesis (MIPO) technique was used. The previous implant was retained in order to minimize surgical trauma and dissection. The patient was operated supine under spinal anaesthesia. The hip was flexed around 30° for adequate reduction and confirmed with an image intensifier.

**Fig. 3:**
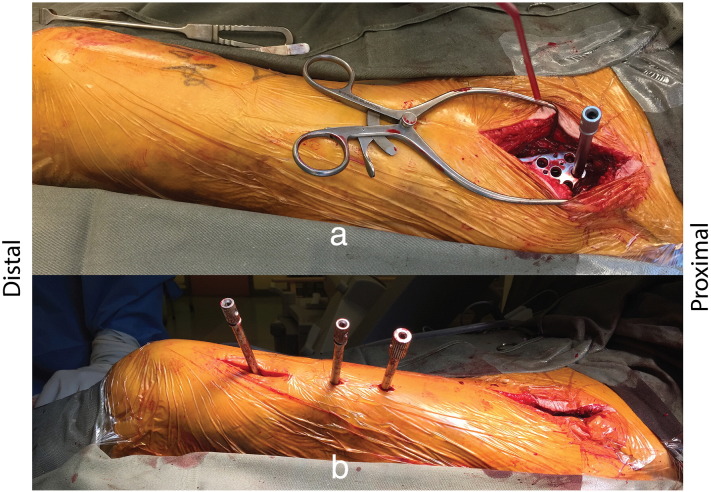
a) Intraoperative photograph showing sub-muscular placement of the plate via a Smith-Peterson approach. b) Distal locking screws were placed though mini-incisions through the anterior quadriceps.

**Fig. 4:**
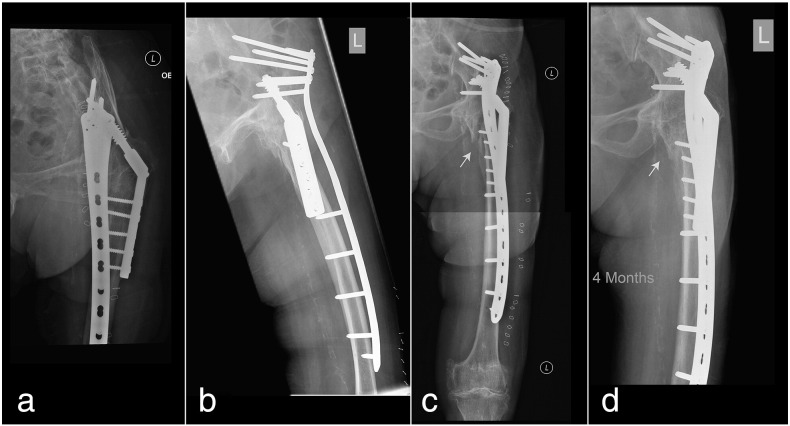
a) Postoperative obturator oblique view showing multiple long locking screws directed towards the supraacetabular ilium. b) Iliac oblique view showing screws superior to the sciatic foramen. c) Post-operative AP view after fixation with presence of a fracture gap. d) Complete union by callus formation at just over 4 months.

A standard Smith-Peterson approach was used to access the ‘hip joint’ region anteriorly (Fig. 3). The pre-templated implant was slid underneath the quadriceps muscle to the anterolateral thigh. A distal split quadriceps incision was created for preliminary placement of K-wires to secure the plate position. Both inlet obturator oblique and inlet iliac oblique views were used to monitor screw placement in the supraacetabular region of the ilium (Fig. 4). Locking screws were placed distally with additional stab incisions. In total, seven locking screws were placed in the proximal fragment and five in the distal fragment. The longest screw in the proximal fragment was 90 mm. Stability of the fixation was ensured and wounds were closed over a suction drain which was removed two days later. The operative blood loss was around 100 ml. The skin to skin operative time was 69 minutes.

The patient was allowed to freely mobilize in bed and full weight bearing walking exercise was started two days after surgery. She was transferred to the rehabilitation unit six days after surgery and further stayed sixteen days there. On discharge, the patient was able to walk with a frame with minor assistance. Her modified Barthel index [13] was 49 out of 100 and Lawton Instrumental Activities of Daily Living scale [14] was 6 out of 27. At two-month follow-up there was evidence of callus and fracture healing with no pain. At four months the fracture has completely united. The patient has returned to her preinjury level of function and continued to walk with a quadripod.

## Discussion

Treatment of a fractured arthrodesed hip is perceived to be challenging because of its rarity, anatomical uncertainty, long lever arm and lack of proximal bone stock especially when associated with osteoporosis. Conversion of fusion to THR has a high complication rate [15], sometimes indicated for healthier patients suffering from mechanically painful adjacent joint and spine conditions [16].

Virtual planning in 3D using reformatted CT or MRI data is useful in surgery for complex and rare deformities [17]. 3D printing brings this further by providing tactile feedback and allowing surgical simulations on near real-life 3D models. Conversely, the limitation of the current technique being the need for CT or MRI imaging and resources required for model preparation. Caution is needed in MIPO, especially when anatomical reduction is desired. Accurate preoperative modeling is more difficult when fragments are severely displaced and unstable because these fragments may not have a consistent spatial relationship at surgery.

This rare problem lacks any case series or comparative studies. Four other reported examples [5], [6], [7], each slightly distinct, told us that successful treatment of a fractured hip arthrodesis is achievable through individualized preoperative planning and stable fixation using a variety of implants.

In this case example, rapid prototyping enabled accurate preoperative planning, implant selection, templating, and avoidance of inference with existing hardware that is not possible with conventional methods. Surgeons were able to carry out this operation safely, rapidly and with minimal surgical trauma. The implant was able to provide a long lever arm of fixation in both fragments which we considered important in osteoporotic bone. With preserved local biology, reasonable fracture reduction and stable bridging fixation, the patient experienced rapid recovery, union and no complications.

## Conflict of interest

No financial or non-financial support has been received by any authors for the published work.

Dr. C Fang reports non-financial support from DePuy Synthes, non-financial support from AO Foundation, the submitted work.

Dr. B Fang has nothing to disclose.

Dr. TW Lau reports grants from DePuy Synthes, non-financial support from AO Foundation, outside the submitted work.

Dr. TM Wong reports grants and non-financial support from AO Foundation, outside the submitted work.

Dr. Pun has nothing to disclose.

Dr. F Leung reports grants, personal fees and non-financial support from DePuy Synthes, grants, personal fees, non-financial support and other from AO Foundation, outside the submitted work.
